# Fantastic Learning Moments and Where to Find Them

**DOI:** 10.5811/westjem.2017.10.35179

**Published:** 2017-12-05

**Authors:** Alexander Y. Sheng, Ryan Sullivan, Kara Kleber, Patricia M. Mitchell, James H. Liu, Jolion McGreevy, Kerry McCabe, Annemieke Atema, Jeffrey I. Schneider

**Affiliations:** * Boston Medical Center, Department of Emergency Medicine, Boston, Massachusetts; † Boston University School of Medicine, Boston, Massachusetts

## Abstract

**Introduction:**

Experiential learning is crucial for the development of all learners. Literature exploring how and where experiential learning happens in the modern clinical learning environment is sparse. We created a novel, web-based educational tool called “Learning Moment” (LM) to foster experiential learning among our learners. We used data captured by LM as a research database to determine where learning experiences were occuring within our emergency department (ED). We hypothesized that these moments would occur more frequently at the physician workstations as opposed to the bedside.

**Methods:**

We implemented LM at a single ED’s medical student clerkship. The platform captured demographic data including the student’s intended specialty and year of training as well as “learning moments,” defined as logs of learner self-selected learning experiences that included the clinical “pearl,” clinical scenario, and location where the “learning moment” occurred. We presented data using descriptive statistics with frequencies and percentages. Locations of learning experiences were stratified by specialty and training level.

**Results:**

A total of 323 “learning moments” were logged by 42 registered medical students (29 fourth-year medical students (MS 4) and 13 MS 3 over a six-month period. Over half (52.4%) intended to enter the field of emergency medicine (EM). Of these “learning moments,” 266 included optional location data. The most frequently reported location was patient rooms (135 “learning moments”, 50.8%). Physician workstations hosted the second most frequent “learning moments” (67, 25.2%). EM-bound students reported 43.7% of “learning moments” happening in patient rooms, followed by workstations (32.8%). On the other hand, non EM-bound students reported that 66.3% of “learning moments” occurred in patient rooms and only 8.4% at workstations (p<0.001).

**Conclusion:**

LM was implemented within our ED as an innovative, web-based tool to fulfill and optimize the experiential learning cycle for our learners. In our environment, patient rooms represented the most frequent location of “learning moments,” followed by physician workstations. EM-bound students were considerably more likely to document “learning moments” occurring at the workstation and less likely in patient rooms than their non EM-bound colleagues.

## INTRODUCTION

**Figure 1:**
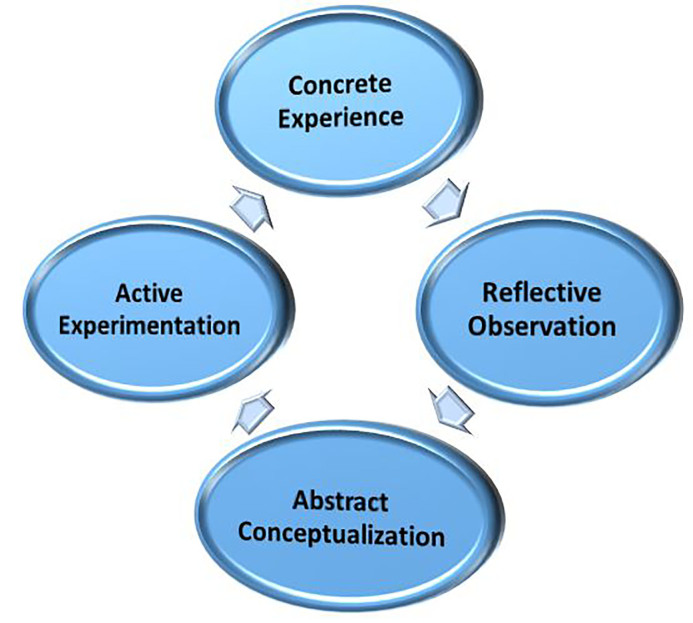
Kolb’s experiential learning cycle.^1^

Experiential learning is critical for successful growth and the development of new skills and behaviors. Kolb’s four-part experiential learning model, which incorporates concrete experience, reflective observation, abstract conceptualization, and active experimentation, can be used in a recurring cycle that supports progressive new learning (Figure 1).^1^ While previous work has discussed these processes as they apply to medical trainees,^2^ there is little literature exploring current mechanisms of information transfer in the modern clinical learning environment. As educators refine their skills to meet the needs of today’s learners, a deeper understanding of exactly where experiential learning occurs will inform medical education theory and practices.

The classic model of bedside teaching has been in decline.^3^ Pressures on academic faculty to care for more patients in less time, and to increase their documentation, billing, and academic productivity, have created often seemingly insurmountable barriers to bedside teaching.^4^ These time pressures are particularly relevant in emergency medicine (EM); while overall faculty-resident interaction time was as high as 20%, direct observation time of residents interacting with patients by faculty was only 3.6%. On the medicine wards, it is as little as 1% of the time.^5^ These data highlight the importance of maximizing learner-educator interactions at the patient’s bedside and elsewhere in the clinical learning environment. Improving our understanding of where such interface occurs is crucial to optimizing them.

**Population Health Research Capsule**

What do we already know about this issue?

Despite playing a crucial role in learner development, there is little literature exploring how and where experiential learning is happening in the modern clinical learning environment.

What was the research question?

We used data from “Learning Moment” (LM) to determine where learning experiences were occuring in our emergency department (ED).

What was the major finding of the study?

Patient rooms represented the most frequent location of “learning moments” in our ED, followed by physician workstations.

How does this improve population health?

Using LM to determine the location of learning experiences has the potential to inform the design of optimal learning ecosystems and maximize experiential learning for future trainees.

We created a novel, web-based educational tool called “*Learning Moment”* (LM) that integrates the principles of asynchronous learning^6^ in order to foster experiential learning. Although most clinical learning environments offer some aspects of Kolb’s learning model (experiences and active experimentation), they rarely provide learners with an organized approach to reflective observation and abstract conceptualization.^1^ LM fulfills these missing elements to help learners learn better at work.

Our goal in this study was to use data captured by LM as a research database to determine where learning experiences were occuring within our emergency department (ED) clinical learning environment. We hypothesized – based on our own experiences and on recent literature (described above) demonstrating that faculty-learner interactions often occur in locations away from the bedside^3^,^5^ – that clinical learning would occur more frequently at physician workstations as opposed to the bedside.

## METHODS

### Intervention

**Figure 2:**
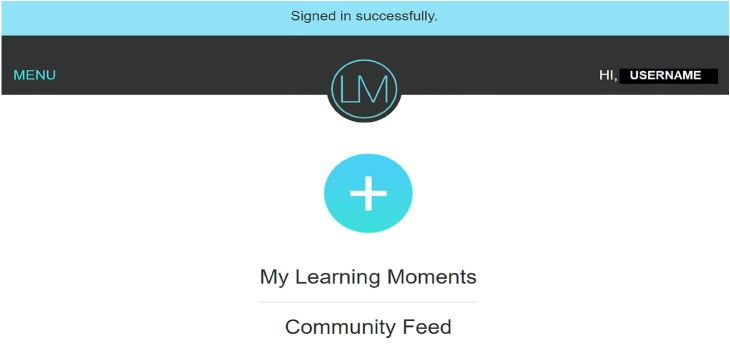
*Learning Moment* interface.

**Figure 3:**
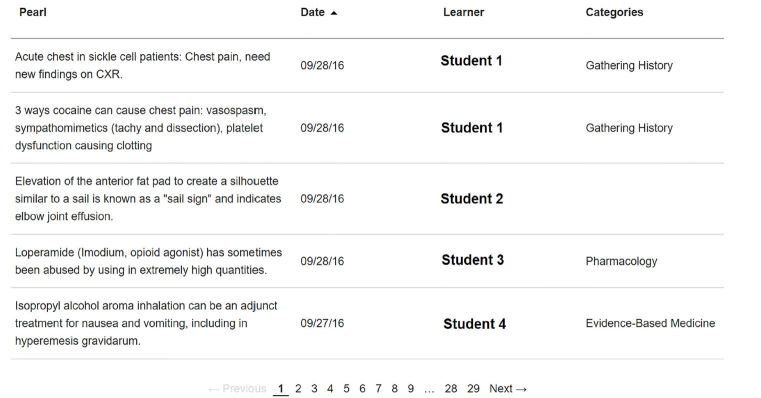
*Learning Moment* Community Feed.

We completed the initial build of LM ( www.learningmoment.org ) to encourage the reflective and abstract conceptualization steps of Kolb’s experience learning model. The LM platform (Figure 2) is designed to serve three main functions:

1. Provide learners with a “note-taking” platform to log learning experiences of their own choosing in the form of “learning moments”. Doing so allows for synthesis of such experiences into coherent thoughts, enhancing understanding and retention through self-reflection.
2. Create a searchable and shareable repository of useful, practical, high-yield educational content in the form of a Community Feed (Figure 3) that benefits our entire learning community: Sharing “learning moments” online and in person provides opportunity for abstract conceptualization.
3. Use the data collected from LM to better understand the current state of experiential learning in the clinical environment – starting with, where does learning occur?

### Implementation

We implemented LM at a busy (130,000 annual ED visits) tertiary care hospital that hosts a postgraduate year 1–4 EM residency with 12 residents per year, as well as robust third- and fourth-year medical student (M3 and M4) clerkships. As part of the monthly student orientation, MS3s and MS4s were introduced to and registered on the LM platform by research assistants. Demographic data, including the student’s intended specialty and year of training were collected at the time of user registration. Students were encouraged to use our platform to log learning experiences during their clerkship without a formal requirement to do so. Students logged self-selected learning experiences in the form of “learning moments” that included the clinical “pearl,” clinical scenario, and location where the “learning moment” occurred, among other optional data variables. Students were encouraged to log onto LM and view the Community Feed to read about “learning moments” logged by their peers.

While peer reflection can promote critical thinking, previous work has stressed the role of faculty facilitators to provide oversight in the reflective process.^7^ Therefore, a faculty review panel consisting of three EM board-certified attendings oversaw the LM website to ensure content validity and Health Insurance Portability and Accountability Act compliance. We held monthly in-person “*Learning Moment* Reflection” sessions led by experienced clinical faculty in small-group format. These sessions provided medical students with additional opportunities to discuss and expand upon the “learning moments” that they had already logged in order to deepen their understanding through further reflection and abstract conceptualization. A link to the LM website was made accessible directly from the electronic medical record to promote ease of access. Our study was approved as exempt by our institutional review board.

### Analysis

We presented data using descriptive statistics with frequencies and percentages, and we stratified locations of learning experiences by specialty and training level. Fisher’s exact test was used to compare the distribution of locations between groups. We used SAS v9.3 (SAS Institute Cary, NC) for all data analysis.

## RESULTS

Of the 53 medical students who completed their EM clerkship rotation, 42 (79.2%) logged at least one “learning moment.” A total of 323 “learning moments” were logged between August 22, 2016 – February 12, 2017, spanning the course of six one-month-long clerkship rotations. The MS group consisted of 29 MS4s and 13 MS3s. Over half (52.4%) intended to enter the field of EM while the remainder were either undecided or intended to train in another specialty (Table 1).

**Figure 4:**
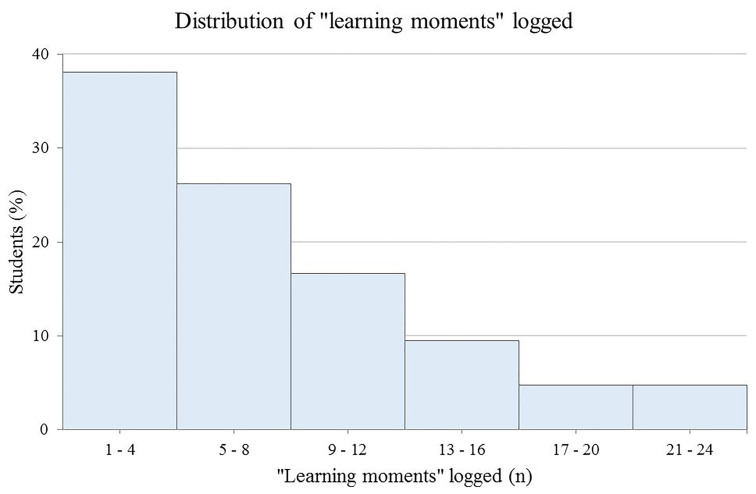
Distribution of number of “learning moments” logged by students.

The median number of “learning moments” logged by these students was six (interquartile ratio=7.5). Nearly 40% (n=16) of the students logged 1–4 “learning moments,” and over 25% (n=11) logged 5–8 “learning moments” (Figure 4).

A total of 266 “learning moments” included optional location data. The most frequently reported location was patient rooms (135 “learning moments,” 50.8%). Physician workstations hosted the second most frequent “learning moments” (67, 25.2%) (Table 2).

The distribution of reported locations of “learning moments” differed between EM-bound and non EM-bound students. EM-bound students reported 43.7% of “learning moments” happening in patient rooms, followed by workstations (32.8%), hallways (14.8), and resuscitation rooms (8.2%). On the other hand, non EM-bound reported 66.3% of “learning moments” having occurred in patient rooms and only 8.4% at workstations (p<0.001).

Differences were also seen in the distribution of “learning moment” location between MS3s and MS4s. MS3s logged 41 (68.3%) “learning moments” happening in patient rooms with the remainder evenly divided between workstations, hallways, and resuscitation rooms. MS4s logged relatively fewer, 94 (45.6%), “learning moments” happening in patient rooms and more, 61 (29.6%), happening at the workstations (p=0.005).

## DISCUSSION

LM is a novel educational platform designed to integrate experiential learning and shared learning. We employed the LM platform to track the location of “learning moments” as a proxy to gain insight into the location of experiential learning in a busy ED. Our data suggest that the majority of “learning moments” occur at the patient bedside, despite our hypothesis to the contrary. By hypothesizing that the majority of “learning moments” on LM would occur away from the bedside, where faculty-learner interactions have been shown to be low,^5^ we may have overemphasized the importance of faculty-student interactions in the learning process. Our results are consistent with Kolb’s notion that the learning is happening everywhere at all times.^1^ In fact, we demonstrated that the majority of “learning moments” were happening without the presence of faculty, but from patient interactions instead.

The decline of bedside teaching and the pressures and barriers influencing it have been well-described.^3^ The findings presented here, however, suggest that it remains an important component of undergraduate medical education. Educators may need to develop new strategies to continue to provide opportunities for bedside teaching for our learners. In addition to existing strategies described in the literature,^3^,^8^ one potential approach already employed at various institutions is to provide dedicated time for faculty teaching shifts or senior resident teaching rotations where education is prioritized over clinical flow and productivity. Increased faculty coverage in the ED may potentially encourage increased time spent directly interacting with learners, especially at the bedside. Furthermore, both resources and time for faculty to attend teach-the-teacher programs would better prepare clinicians to be competent educators in order to maximize bedside teaching opportunities that currently exist. Faculty incentives such as teaching awards, financial reimbursements, and promotion may further encourage bedside teaching.

The results of this study indicate that the physician workstation is the most common non-bedside location of “learning moments,” especially for EM-bound students. It is likely of benefit to optimize educational experiences in this setting as well. Multiple potential strategies to teach effectively at workstations have been described in the literature.^8^,^9^ Additional ways to take advantage of learning interactions at the workstation include making available a collection of cases, images, electrocardiograms etc. on a shared drive for when teachable moments arise. Students often use online resources to answer questions during clinical work. It is essential to provide links to reliable resources for evidence-based medicine, clinical guidelines, and clinical decision-rules. At the same time, medical schools should continue to train students to be thoughtful and efficient curators and interpreters of literature. Interventions to optimize experiential learning at the bedside and at the workstation could potentially be directly evaluated using LM to see if more “learning moments” are logged as a result.

Interestingly, EM-bound students reported notably fewer “learning moments” occurring in patient rooms and more occurring at the workstations than their non EM-bound colleagues. These findings raise the question: Are there inherent differences between the learning preferences of EM-bound students vs. non EM-bound students? Considering that aversion to bedside rounding is commonplace among EM residents, one could postulate that those who choose EM as their intended specialty may have an increased propensity to learn “on the spot,” such as at the workstations where presentations of cases commonly occur. It is conceivable that EM-bound students may preferentially receive more teaching from residents and faculty who are aware of their learners’ decision to enter EM. The shift in location of “learning moments” from bedside to workstations from the MS3 to MS4 year may represent changing learning preferences as students progress along the spectrum from novice to expert learners, or may simply be the result of a large proportion of rotating MS4s who chose EM as their intended specialty compared to mostly undecided MS3s (Table 1). It would be valuable to conduct a similar study in EM resident populations to see if the results differ; such insight could potentially benefit graduate medical education training.

## LIMITATIONS

Our study has several limitations. We emphasized during orientation and registration to LM that the “location” drop-down menu when logging a “learning moment” on the website was meant to indicate the location of where the “learning moment” occurred, not the location of the patient. Students may still have mistakenly chosen the patient’s room number when they logged some “learning moments.” Doing so would have falsely elevated the number of “learning moments” documented in patient rooms. Furthermore, out of 323 total “learning moments”, only 266 contained the optional location data. Our study is limited by the use of learner self-selected learning experiences in the form of “learning moments” as an imperfect proxy to gain insight to the location of experiential learning in the ED environment. No direct observation of where learning was happening was performed. Recall bias is a significant confounder to our data as we intentionally left it up to the students to choose what learning experiences to log as a “learning moment.” Certain experiences may be overlooked when learners fail to consciously notice that learning is happening. They may also choose to document certain types of learning experiences over others. Lastly, our pilot was conducted at a single department within one institution. Other learning environments may yield different results. Nevertheless, data from LM has the potential to help educators better understand the intricacies of local learning microenvironments as well as the broader clinical learning ecosystem.

## CONCLUSION

LM was implemented within our ED as an innovative, web-based tool to fulfill and optimize the experiential learning cycle for our learners. In our environment, patient rooms represented the most frequent location of “learning moments,” followed by physician workstations. EM-bound students were considerably more likely to document “learning moments” occurring at the workstation and less likely in patient rooms than their non EM-bound colleagues. Although successfully piloted in the ED, LM is potentially adaptable to other clinical departments and institutions as we seek to inform the design of optimal learning ecosystems and maximize experiential learning for all future trainees. Efforts are ongoing to make LM available to more learner populations in new learning environments as we continue to demonstrate the feasibility and value of our platform to various stakeholders throughout health professions education.

## Figures and Tables

**Table 1:** Characteristics of medical students who participated in *Learning Moment.*

Characteristics	N=42
Training level, n (%)	
MS4	29 (69.0)
MS3	13 (31.0)
Intended specialty, n (%)	
EM	22 (52.4)
Other/undecided	20 (47.6)
Logged “learning moment” per student, median (IQR)	6 (7.5)

*EM*, emergency medicine, *MS*, medical student.

**Table 2:** Location of “learning moments” by intended specialty and training level (n=266).

		Intended specialty^1^		Training level^2^	
			
Location	Total (%)	EM	Other/undecided	MS4	MS3
Hallway	39 (14.7)	25 (13.7)	14 (16.9)	33 (16.0)	6 (10.0)
Pharmacy	1 (0.4)	1 (0.6)	0 (0.0)	1 (0.5)	0 (0.0)
Patient room	135 (50.8)	80 (43.7)	55 (66.3)	94 (45.6)	41 (68.3)
Resuscitation hallway	2 (0.8)	2 (1.1)	0 (0.0)	2 (1.0)	0 (0.0)
Resuscitation room	22 (8.3)	15 (8.2)	7 (8.4)	15 (7.3)	7 (11.7)
Workstation	67 (25.2)	60 (32.8)	7 (8.4)	61 (29.6)	6 (10.0)

*EM*, emergency medicine, *MS*, medical student.

1 Fisher’s exact p-value<0.001;

2 Fisher’s exact p-value=0.005

## References

[1] Kolb David (1984). Experiential Learning: Experience as the Source of Learning and Development.

[2] Armstrong E, Parsa-Parsi R (2005). How can physicians’ learning styles drive educational planning?. Acad Med.

[3] Aldeen AZ, Gisondi MA (2006). Bedside teaching in the emergency department. Acad Emerg Med.

[4] Cooke M, Irby DM, Sullivan W (2006). American medical education 100 years after the Flexner Report. N Engl J Med.

[5] Chisholm CD, Whenmouth LF, Daly EA (2004). An evaluation of emergency medicine resident interaction time with faculty in different teaching venues. Acad Emerg Med.

[6] Mayadas F (1997). Asynchronous learning networks: A Sloan Foundation perspective. J Asynchronous Learn Netw.

[7] Bernard AW, Gorgas D, Greenberger S (2012). The use of reflection in emergency medicine education. Acad Emerg Med.

[8] Green GM, Chen EH (2015). Top 10 ideas to improve your bedside teaching in a busy emergency department. Emerg Med J.

[9] Irby DM, Wilkerson L (2008). Teaching when time is limited. BMJ.

